# Epidemiological characteristics of HIV transmission in southeastern China from 2015 to 2020 based on HIV molecular network

**DOI:** 10.3389/fpubh.2023.1225883

**Published:** 2023-10-18

**Authors:** Zhenghua Wang, Dong Wang, Liying Lin, Yuefeng Qiu, Chunyan Zhang, Meirong Xie, Xiaoli Lu, Qiaolin Lian, Pingping Yan, Liang Chen, Yi Feng, Hui Xing, Wei Wang, Shouli Wu

**Affiliations:** ^1^Fujian Provincial Center for Disease Control and Prevention, Fujian Provincial Key Laboratory of Zoonosis Research, Fuzhou, China; ^2^Chinese Center for Disease Control and Prevention, Beijing, China; ^3^Fuzhou Institute for Disease Control and Prevention of China Railway Nanchang Bureau Group Co., Ltd., Fuzhou, China; ^4^National Health Commission Key Laboratory for Parasitic Disease Prevention and Control, Jiangsu Provincial Key Laboratory for Parasites and Vector Control Technology, Jiangsu Institute of Parasitic Diseases, Wuxi, China; ^5^School of Public Health, Fujian Medical University, Fuzhou, China

**Keywords:** HIV/AIDS, molecular network, epidemiological characteristic, southeastern China, drug resistance

## Abstract

**Objective:**

HIV/AIDS remains a global public health problem, and understanding the structure of social networks of people living with HIV/AIDS is of great importance to unravel HIV transmission, propose precision control and reduce new infections. This study aimed to investigate the epidemiological characteristics of HIV transmission in Fujian province, southeastern China from 2015 to 2020 based on HIV molecular network.

**Methods:**

Newly diagnosed, treatment-naive HIV/AIDS patients were randomly sampled from Fujian province in 2015 and 2020. Plasma was sampled for in-house genotyping resistance test, and HIV molecular network was created using the HIV-TRACE tool. Factors affecting the inclusion of variables in the HIV molecular network were identified using univariate and multivariate logistic regression analyses.

**Results:**

A total of 1,714 eligible cases were finally recruited, including 806 cases in 2015 and 908 cases in 2020. The dominant HIV subtypes were CRF01_AE (41.7%) and CRF07_BC (38.3%) in 2015 and CRF07_BC (53. 3%) and CRF01_AE (29.1%) in 2020, and the prevalence of HIV drug resistance was 4.2% in 2015 and 5.3% in 2020. Sequences of CRF07_BC formed the largest HIV-1 transmission cluster at a genetic distance threshold of both 1.5 and 0.5%. Univariate and multivariate logistic regression analyses showed that ages of under 20 years and over 60 years, CRF07_BC subtype, Han ethnicity, sampling in 2015, absence of HIV drug resistance, married with spouse, sampling from three cities of Jinjiang, Nanping and Quanzhou resulted in higher proportions of sequences included in the HIV transmission molecular network at a genetic distance threshold of 1.5% (*p* < 0.05).

**Conclusion:**

Our findings unravel the HIV molecular transmission network of newly diagnosed HIV/AIDS patients in Fujian province, southeastern China, which facilitates the understanding of HIV transmission patterns in the province.

## Introduction

Following concerted efforts for over four decades, HIV remains a global public health problem (1). Currently, HIV transmission via blood, injection drug use or mother-to-child has been almost under effective control (2); however, there is no remarkable decline in the annual global number of HIV/AIDS cases (3). A recent study reported that the global age-standardized HIV/AIDS prevalence, death, and disability adjusted life years (DALYs) rate in 2019 increased by 307.26, 4.34, and 221.91 per 100,000 cases in relative to in 1990 (4). More importantly, resurgence of HIV/AIDS has been detected across the world (5, 6). Ending AIDS as a public health threat by 2030 is increasingly under a great challenge (7). Understanding the structure of social networks of people living with HIV/AIDS is of great importance to unravel HIV transmission, propose precision control and reduce new infections (8, 9).

Recently, characterization of HIV-1 transmission with the gene sequences from routine monitoring of HIV drug resistance based on molecular network analysis has been performed for identification of people at a high risk of transmission and implementation of immediate interventions (10–12). A recent molecular transmission network-based study identified young people outside of school as the main high-risk group for spreading HIV to students and the key group leading to the spread of HIV among young students in Guangxi, southern China (13). In addition, molecular transmission network analyses targeting older adults revealed that commercial sexual behaviors between older adult men and female sex workers led to the rapid spread of HIV in Zhejiang province, eastern China and Guangxi, southern China (14, 15). Based on molecular networks and spatial epidemiology, a network connectivity analysis showed that some geographically distant cities of Sichuan province had stronger transmission links than cities that were closer together, indicating that molecular monitoring is more effective to identify geographically dispersed propagation clusters (16). To investigate the epidemic characteristics of virus strains in various cities of Zhejiang province, a molecular network analysis was performed and Jiaxing City was found to have geographical clustering and a certain number of HIV/AIDS patients with high transmission risk (17). In the current study, a molecular transmission network analysis of HIV/AIDS was performed, based on monitoring of HIV drug resistance, aiming to rapid identification of individuals with high-risk transmission and at high risk of HIV infection in Fujian province, southeastern China, and to reduce new HIV infection with immediate targeted interventions.

## Materials and methods

### Study subjects

All newly diagnosed HIV/AIDS patients were sampled from Fujian province in 2015 and 2020, and all participants were naïve for antiretroviral therapy. Participants’ demographics, route of HIV infection, HIV subtype and clinical symptoms were collected from medical records and individual investigation forms.

### In-house genotyping resistance test

10 mL EDTA-anticoagulated blood was sampled from each participant, centrifuged within 6 h post-sampling, and the plasma was collected. Viral RNA was extracted from plasma using QIAamp Viral RNA Mini Kits (QIAGEN GmbH; Hilden, Germany), and subjected to reverse transcriptional amplification using in-house genotyping resistance test with the primers for implication of HIV subtypes CRF07-BC and B, which are effective to detect HIV-1 subtype A1, B, C, D, G, CRF01_AE, CRF02_AG, CRF06_cpx, CRF07_BC, CRF08_BC, CRF15_01B and other recombinant strains (18) (Table 1). The amplification products were purified and sequenced by Sangon Biotech (Shanghai, China). The fragments of amplified and sequenced target genes covered 4–99 amino acids in the protease region and 38 to 320 amino acids in the reverse transcriptase region.

**Table 1:** Sequences of primers for amplification of HIV subtypes CRF07-BC and CRF07-B.

Primer	Sequence of primer for HIV subtype CRF07-BC (5′-3′)		Sequence of primer for HIV subtype CRF07-B (5′-3′)	
Amplification primer	RT20	CTGCCAATTCTAATTCTGCTTC	RT20	CTGCCAGTTCTAGCTCTGCTTC
	PRO1	CAGAGCCAACAGCCCCACCA	PRO1	CAGAGCCAACAGCCCCACCA
	MAW26	TGGAAATGTGGAAAAGAAGGAC	MAW26	TTGGAAATGTGGAAAGGAAGGAC
	RT21	CTGTATTTCAGCTATCAAGTCTTTTGATGGG	RT21	CTGTATTTCTGCTATTAAGTCTTTTGATGGG
Sequencing primer	PRO1	CAGAGCCAACAGCCCCACCA	PROS3	GCCAACAGCCCCACCA
	RTAS-qian	GGACCTACACCTGTCAAC	RTAS-qian	GGACCTACACCTGTCAAC
	RTB	CCTAGTATAAACAATGAGACAC	RTB	CCTAGTATAAACAATGAGACAC
	PROC1S	GCTGGGTGTGGTATTCC	PROC1S	GCTGGGTGTGGTATTCC
	RT20-07 BC	CTGCCAATTCTAATTCTGCTTC	RT20S3	GTTCTAGCTCTGCTTC

### Analysis of HIV drug resistance

The sequences were subjected to quality assessment, editing, assembly and mixed-base calling using the software ChromasPro version 2.4.1 and BioEdit version 7.2, and then uploaded to the Stanford HIV Drug Resistance Database (https://hivdb.stanford.edu/) for analysis of HIV drug resistance.

### Generation of HIV molecular network

Due to the sampling time span of 5 years in this study, a molecular network was constructed using a 1.5% gene distance threshold, while a molecular network at a 0.5% gene distance threshold was created to highlight recent HIV infections and merge with the molecular network at a 1.5% gene distance threshold. All gene sequences with length of 1,000 bp and longer and mixed-base proportion of <5% were aligned and saved as .fas files, and all epidemiological data corresponding to each sequence were saved as .csv files. Pairwise genetic distances were estimated using the Tamura-Nei 93 (TN93) fast distance calculator, and HIV molecular network was created using the HIV-TRACE tool (19). All nodes in the HIV molecular network were assigned with epidemiological data, and molecular network maps were generated.

### Statistical analysis

All data were entered into Microsoft Excel 2010 (Microsoft Corporation; Redmond, WA, United States). Differences of proportions were tested for statistical significance with chi-square test. Factors affecting the inclusion of variables in the HIV molecular network were identified using univariate and multivariate logistic regression models with the inclusion of sequence in the molecular cluster as a dependent variable and age, gender, molecular subtype, educational level, ethnicity, sampling time, route of HIV transmission, HIV drug resistance, marital status and sampling regions as independent variables. All statistical analyses were performed using the software SPSS version 22.0 (SPSS, Inc.; Chicago, IL, United States), and a *p* value of <0.05 was considered statistically significant.

### Ethical statement

This study was approved by the Medical Ethics Review Committee of Fujian Provincial Center for Disease Control and Prevention (approval no. 2019–013). All procedures and performed following Declaration of Helsinki, as well as international and national laws, regulations and guidelines for human studies.

## Results

### Subject characteristics

A total of 1,858 treatment-naïve, newly diagnosed HIV/AIDS cases were enrolled, including 922 cases in 2015 and 936 cases in 2020. If samples with partial negative amplification and partial successful amplification with sequence length of 1,000 bp and shorter and/or mixed base of >5%, a total of 1,714 cases were included in the final analysis, including 806 cases in 2015 and 908 cases in 2020. Most cases were men (84.31%), and the highest number of cases was seen at ages of 20 to 29 years (28.35%). Sexual contact was the predominant route of HIV transmission (96.27%), and illiteracy and primary school was the main educational level (32.56%) (Table 2).

**Table 2:** Subject characteristics.

Characteristic		Number (proportion)		
		2015 (*n* = 806)	2020 (*n* = 908)	Total (*n* = 1714)
Age (years)	< 20	39 (4.85)	28 (3.08)	67 (3.91)
	20 to 29	285 (35.36)	201 (22.14)	486 (28.35)
	30 to 39	180 (22.33)	156 (17.18)	336 (19.61)
	40 to 49	150 (18.61)	140 (15.42)	290 (16.92)
	50 to 59	82 (10.17)	200 (22.03)	282 (16.45)
	60 and older	70 (8.68)	183 (20.15)	253 (14.76)
Gender	Male	682 (84.62)	763 (84.03)	1,445 (84.31)
	Female	124 (15.38)	145 (15.97)	269 (15.69)
HIV subtype	CRF07_BC	309 (38.33)	484 (53.30)	793 (46.27)
	CRF01_AE	336 (41.69)	265 (29.19)	601 (35.06)
	B	49 (6.08)	20 (2.20)	69 (4.03)
	C	2 (0.25)	20 (2.20)	22 (1.28)
	CRF55_01B	48 (5.96)	30 (3.30)	78 (4.55)
	CRF08_BC	28 (3.47)	42 (4.63)	70 (4.08)
	Others	34 (4.22)	47 (5.18)	81 (4.73)
Educational level	Illiteracy and primary school	207 (25.68)	351 (38.65)	558 (32.56)
	Junior high school	210 (26.05)	162 (17.84)	372 (21.70)
	Senior high school or vocational secondary school	231 (28.66)	275 (30.29)	506 (29.52)
	Junior college	154 (19.11)	116 (12.78)	270 (15.75)
	Unknown	4 (0.50)	4 (0.44)	8 (0.47)
Ethnicity	Han	790 (98.01)	868 (95.60)	1,658 (96.73)
	Others	16 (1.99)	35 (3.85)	51 (2.98)
	Unknown	0 (0)	5 (0.55)	5 (0.29)
Route of HIV transmission	Heterosexual transmission	457 (56.70)	605 (66.63)	1,062 (61.95)
	Men who have sex with men transmission	341 (42.31)	247 (27.20)	588 (34.31)
	Others	8 (0.99)	53 (5.84)	61 (3.56)
	Unknown	0 (0)	3 (0.33)	3 (0.18)
Marital status	Divorced or widowed	94 (11.66)	147 (16.19)	241 (14.07)
	Married with spouse	351 (43.55)	426 (46.92)	777 (45.33)
	Unmarried	361 (44.79)	331 (36.45)	692 (40.37)
	Unknown	0 (0)	4 (0.44)	4 (0.23)

### Trends in HIV subtype

Among totally 1,714 sequences, the predominant HIV subtypes were CRF07_BC (46.2%) and CRF01_AE (35.0%), and the dominant HIV subtypes were CRF01_AE (41.7%) and CRF07_BC (38.3%) in 2015 and CRF07_BC (53. 3%) and CRF01_AE (29.1%) in 2020. The proportion of the HIV CRF01_AE genotype reduced by 12.6% (*χ*^2^ = 5.5, *p* < 0.000,1) and the proportion of the HIV CRF07_BC genotype increased by 15% (*χ*^2^ = 23.9, *p* < 0.000,1) in 2020 relative to in 2015.

### HIV drug resistance

The prevalence of HIV drug resistance was 4.2% in 2015 and 5.3% in 2020, and a higher prevalence rate of non-nucleoside reverse transcriptase inhibitors (NNRTIs) resistance was seen than that of protease inhibitor and nucleoside reverse transcriptase inhibitor (NRTI) resistance in both 2015 and 2020 (Table 3).

**Table 3:** HIV drug resistance in 2015 and 2020.

Drug resistance rate	2015	2020	Total
Rate of protease inhibitor resistance	0.1% (1/806)	0.1% (1/908)	0.1% (2/1,714)
Rate of nucleoside reverse transcriptase inhibitor resistance	1.1% (9/806)	1.1% (10/908)	1.1% (19/1,714)
Rate of non-nucleoside reverse transcriptase inhibitors	3.0% (24/806)	4.8% (44/908)	4.0% (68/1,714)
Overall drug resistance rate	4.2% (34/806)	5.3% (48/908)	4.8% (82/1,714)

### HIV molecular network characteristics

**Figure 1:**
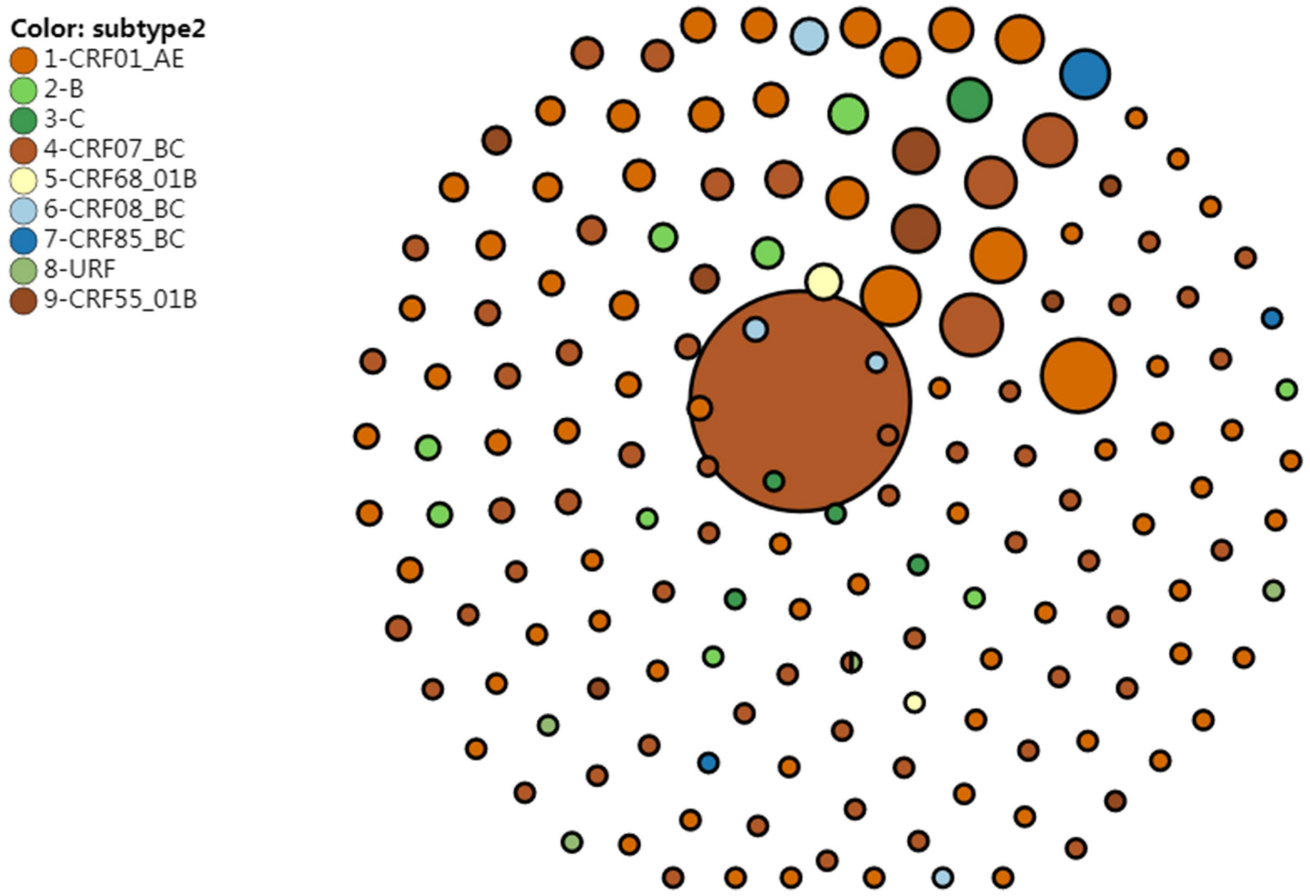
HIV molecular network reveals HIV transmission clusters at a genetic distance threshold of 0.5 and 1.5%.

A genetic distance threshold of 1.5% was used to create the HIV molecular network, and a HIV molecular network was generated at a genetic distance threshold of 0.5% for identifying recent HIV infections, which was merged with the HIV molecular network created at a genetic distance threshold of 1.5%. A total of 50.6% sequences were included in the HIV molecular network at a genetic distance threshold of 1.5%, which generated 162 HIV transmission clusters, and sequences of CRF07_BC formed the largest HIV-1 transmission cluster, which contained 272 nodes. Sequences of CRF01_AE formed the second largest HIV-1 transmission cluster, which contained 30 nodes, and there were 30 HIV transmission clusters that contained more than 4 nodes. At a genetic distance threshold of 0.5%, a total of 17.4% sequences were included in the HIV molecular network. Sequences of CRF07_BC formed the largest HIV-1 transmission cluster, which contained 11 nodes, and there were 12 HIV transmission clusters that contained more than 4 nodes (Figure 1).

Univariate analysis showed significant differences in the proportion of sequences included in the HIV transmission molecular network in terms of age groups, HIV subtype, emergence of HIV drug resistance and marital status at a 1.5% gene distance threshold (*p* < 0.05). Multivariate logistic regression analysis showed that ages of under 20 years and over 60 years, CRF07_BC subtype, Han ethnicity, sampling in 2015, absence of HIV drug resistance, married with spouse, sampling from JJ, NP, and QZ resulted in higher proportions of sequences included in the HIV transmission molecular network (*p* < 0.05) (Table 4).

**Table 4:** Univariate and multivariate analyses of factors affecting the inclusion of sequences in the HIV molecular network.

Variable		No. included in the molecular network (proportion)	Univariate analysis		Multivariate logistic regression analysis	
			*OR* (95% *CI*)	*p* value	*OR* (95% *CI*)	*p* value
Age (years)	< 20	44 (65.6%)	2.107 (1.21, 3.668)	0.008,4	2.812 (1.492, 5.299)	0.001,4
	20 to 29	240 (49.3%)	1.075 (0.803, 1.438)	0.628,1		
	30 to 39	161 (47.9%)	1.013 (0.74, 1.388)	0.934,2		
	40 to 49	138 (47.5)	Reference			
	50 to 59	144 (51.0%)	1.490 (0.828, 1.596)	0.405,7		
	60 and older	141 (55.7%)	1.387 (0.988, 1.945)	0.058,5	1.549 (1.078, 2.226)	0.018
Gender	Male	739 (51.1%)	1.136 (0.825, 1.474)	0.337,5		
	Female	129 (47.9%)	Reference			
HIV subtype	CRF07_BC	455 (57.3%)	1.845 (1.419, 2.399)	0.005,4	1.981 (1.492, 2.63)	<0.000,1
	CRF01_AE	278 (46.2%)	1.179 (0.897, 1.551)	0.237,3		
	Others	135 (42.1%)	Reference			
Educational level	Illiteracy and primary school	293 (52.5%)	1.245 (0.957, 1.619)	0.102,6		
	Junior high school	254 (50.2%)	1.135 (0.868, 1.483)	0.355,6		
	Senior high school or vocational secondary school	141 (52.2%)	1.230 (0.899, 1.684)	0.195,3		
	Junior college and above	175 (47%)	Reference			
	Unknown	5 (62.5%)	1.876 (0.442, 7.964)	0.393,6		
Ethnicity	Han	789 (51.2%)	2.295 (1.261, 4.179)	0.006,6	2.140 (1.144, 4.004)	0.017,3
	Others	16 (31.4%)	Reference			
	Unknown	3 (60%)	3.28 (0.498, 21.593)	0.216,6		
Sampling time	2015	478 (53.1%)	1.204 (0.996, 1.456)	0.055,2	1.421 (1.138, 1.775)	0.001,9
	2020	440 (48.5%)	Reference			
Route of HIV transmission	Heterosexual transmission	527 (49.6%)	Reference			
	Men who have sex with men transmission	312 (53.1%)	1.148 (0.938, 1.404)	0.181,1		
	Others	29 (47.5%)	0.920 (0.549, 1.542)	0.751,8		
	Unknown	0	0			
HIV drug resistance	Yes	17 (20.7%)	0.240 (0.140, 0.413)	< 0.000,1	0.253 (0.144, 0.446)	< 0.000,1
	No	851 (52.1%)	Reference			
Marital status	Divorced or widowed	103 (42.7%)	Reference			
	Married with spouse	415 (53.4%)	1.536 (1.148, 2.056)	0.003,9	1.619 (1.192, 2.198)	0.002
	Unmarried	349 (50.4%)	1.363 (1.014, 1.832)	0.039,9		
	Unknown	1 (25.0%)	0.448 (0.046, 4.36)	0.488,9		
Sampling regions	Fuzhou city	205 (50.6%)	1.330 (0.868, 2.04)	0.190,6		
	Jinjiang city	23 (65.7%)	2.487 (1.123, 5.508)	0.024,6	2.873 (1.252, 6.593)	0.012,8
	Longyan city	26 (40.6%)	0.888 (0.474, 1.663)	0.710,6		
	Ningde city	47 (43.5%)	Reference			
	Nanping city	44 (66.7%)	2.595 (1.372, 4.911)	0.003,4	3.738 (1.91, 7.318)	0.000,1
	Putian city	34 (43.0%)	0.981 (0.546, 1.761)	0.947,8		
	Quanzhou city	234 (56.1%)	1.66 (1.083, 2.543)	0.02	1.728 (1.107, 2.698)	0.016,1
	Sanming city	49 (53.3%)	1.479 (0.846, 2.587)	0.17		
	Xiamen city	163 (44.7%)	1.047 (0.679, 1.614)	0.834,2		
	Zhanzhou city	43 (51.8%)	1.395 (0.785, 7.478)	0.255,9		

**Figure 2:**
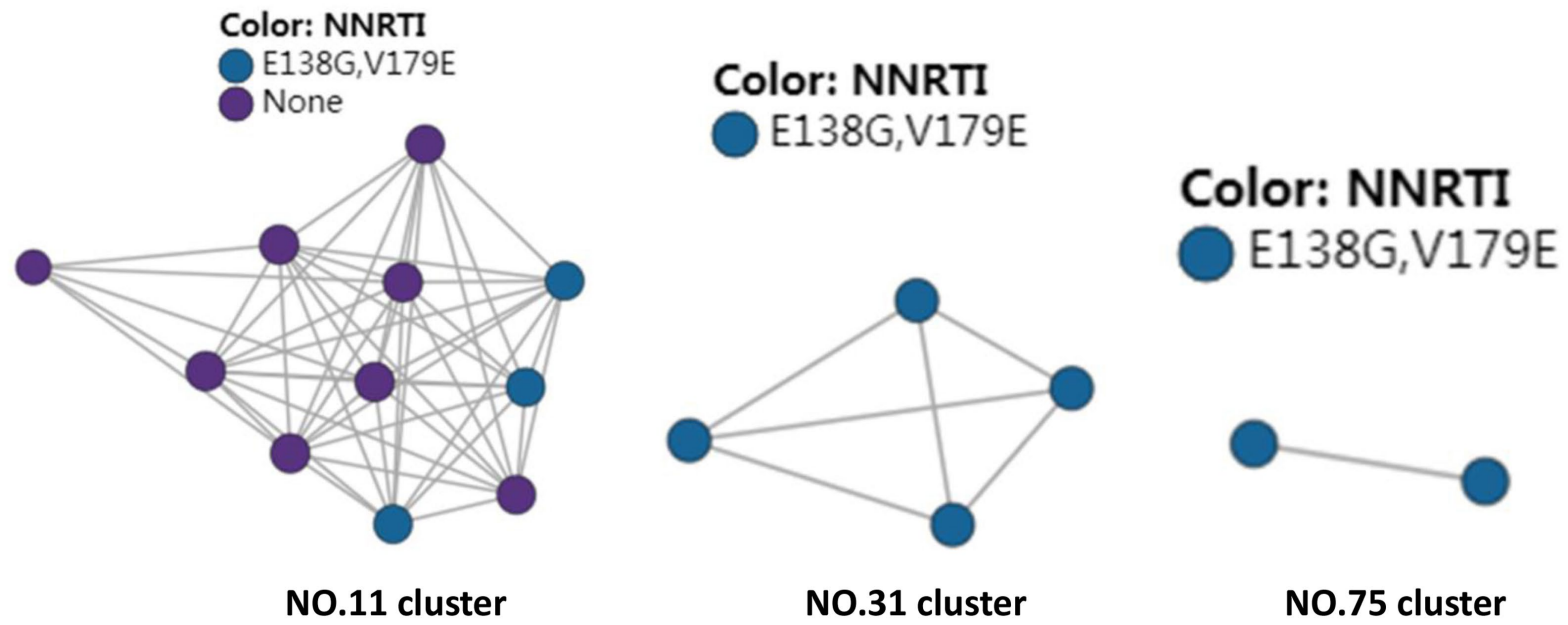
HIV molecular network identifies transmission clusters responsible for HIV drug resistance.

There were three transmission clusters (11, 31, and 75) including sequences with DR mutations for HIV drug resistance, in the created HIV molecular network. These three clusters all carried E138G and V189E, which led to NNRTIs resistance. These three clusters, which contained 11, 4, and 2 nodes, were all formed by CRF55_01B subtype, and patients carrying these sequences were all men and predominantly single (Figure 2).

**Figure 3:**
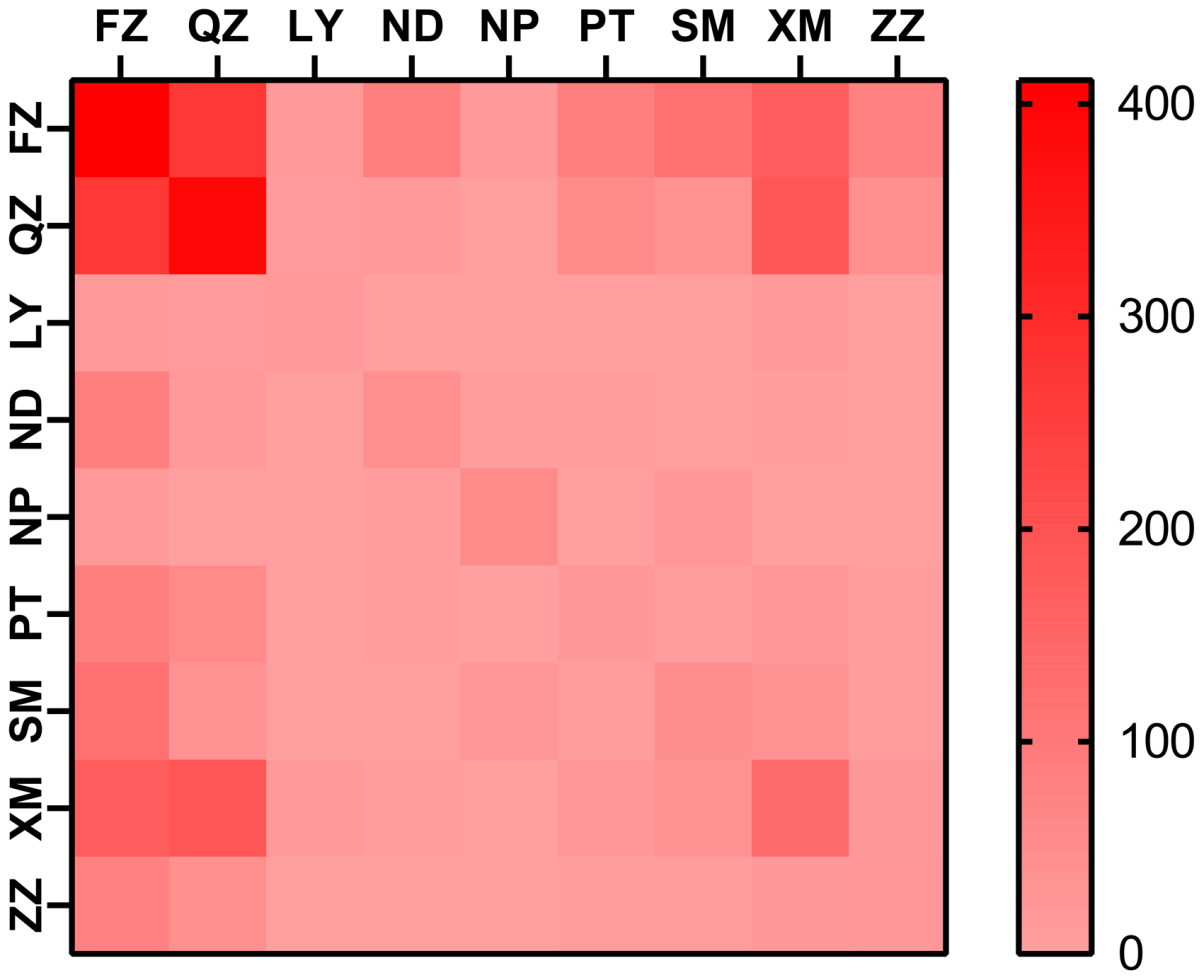
Heatmap for intraregional HIV molecular transmission network in Fujian province. FZ, Fuzhou city; LY, Longyan city; ND, Ningde city; NP, Nanping city; PT, Putian city; QZ, Quanzhou city; SM, Sanming city; XM, Xiamen city; ZZ, Zhangzhou city.

**Figure 4:**
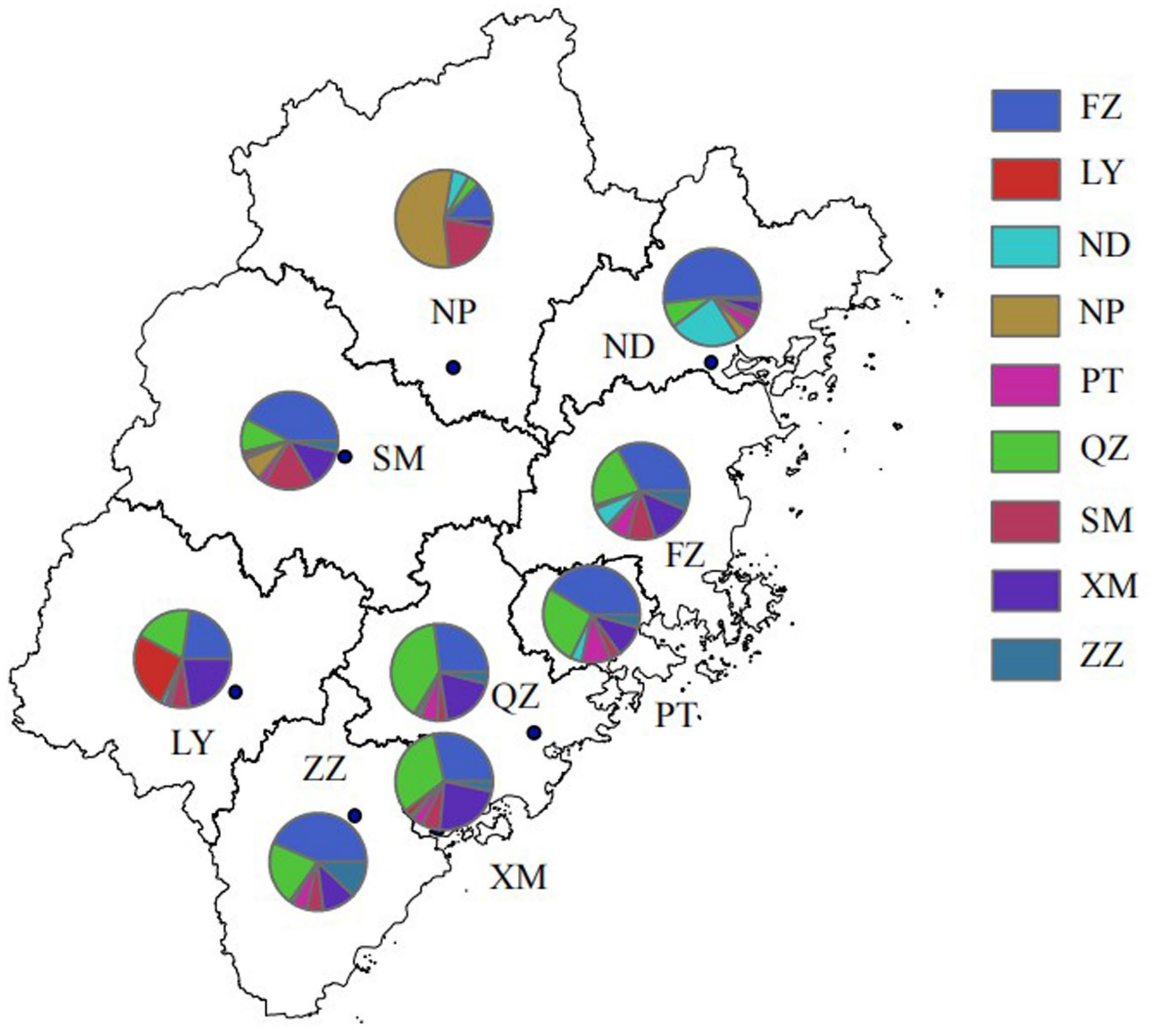
Intraregional HIV molecular network in Fujian province. FZ, Fuzhou city; LY, Longyan city; ND, Ningde city; NP, Nanping city; PT, Putian city; QZ, Quanzhou city; SM, Sanming city; XM, Xiamen city; ZZ, Zhangzhou city.

At a genetic distance at 1.5% gene distance threshold, there were 60.7% nodes with heterosexual transmission as the route of HIV transmission (527/868), including 402 males and 125 females and 35.9% with MSM as the route of HIV transmission (312/868) (Table 5), and there were 27% nodes with sampling sites in Quanzhou city (234/868), 23.6% in Fuzhou city (205/868) and 18.8% in Xiamen City (163/868) (Figure 3). A pie chart was created to display intraregional molecular networking, and the major connections in Nanping city (an inland region) came from local regions, suggesting that intraregional HIV transmission was predominant, while the HIV transmission in Longyan city (an inland region) was closely connected with three cities of Quanzhou, Fuzhou and Xiamen, and the HIV transmission in Sanming and Ningde cities was strongly connected with Fuzhou city. Molecular networking showed a high proportion of connections between Fuzhou city (capital of Fujian province) and cities along the coastal regions in Fujian province, which may be attributed to spatial proximity and convenient transportation. In addition, molecular networking revealed that the connections between Quanzhou city and other cities in Fujian province gradually reduced with the spatial distance, which may be attributed to spatial proximity (Figure 4).

**Table 5:** Connectivity of nodes in the HIV molecular network specified by route of HIV transmission.

Route of transmission	Hex	IDU	MCT	MSM	OTH	SI
Hex	1770	2	4	1,013	67	2
IDU	2	0	0	1	0	0
MCT	4	0	0	0	0	0
MSM	1,013	1	0	988	11	2
Oth	67	0	0	11	6	0
SI	2	0	0	2	0	0

Hex, heterosexual transmission; IDU, injection drug use; MCT, mother-to-child transmission; MSM, men who have sex with men; Oth, others; SI, sexual transmission plus injection drug use.

## Discussion

Social network of HIV-infected individuals is of critical importance for HIV transmission (15–17). Therefore, understanding the social network of HIV-infected individuals is of great significance to unravel HIV transmission patterns, propose targeted and precision interventions and reduce new infections (20–22). Because of latent HIV infection (23), timing from HIV infection to diagnosis (24), a low possibility of acquiring HIV infection from a single high-risk behaviors (25), and difficulty in acquiring accurate sexual or drug use behaviors through traditional epidemiological surveys (26), conventional epidemiological approaches, such as history of infection contacts and traceability investigation, are difficult for analysis of HIV transmission networks, while molecular epidemiology is effective to supplement the shortcomings of conventional epidemiology and social network analysis (27, 28).

The development of molecular epidemiological approaches and highly efficient replication and low-fidelity reverse transcription of HIV-1 lead to high similarity but non-identity between progeny and parental viruses (29). Therefore, estimating the genetic distance between sequences from different infected individuals may be useful to identify the potential transmission pattern among HIV-infected cases that sequences corresponded to (30). It has been reported that HIV transmission network analysis using gene sequences from routine monitoring of HIV drug resistance based on molecular network is effective to guide the formulation of the AIDS control strategy (10–12), and HIV transmission network analysis has been included as one of the national control strategies for new HIV infections (31).

Currently, the common molecular network analysis approaches include pairwise genetic distance estimation, phylogenetic reconstruction alone and in combination, each approach has its advantages and disadvantages. The Cluster Picker tool is effective to identify the cluster that contains only two sequences, while the HIV-TRACE tool appears to identify large or small clusters. In this study, the genetic distance in the HIV molecular network was estimated with the TN93 fast distance calculator using the HIV-TRACE tool (32).

This is the first study to create the HIV molecular transmission network of newly diagnosed HIV/AIDS patients in Fujian province, southeastern China. At a genetic distance threshold of 1.5%, a total of 162 HIV transmission clusters were generated, with 30 transmission clusters containing more than 4 nodes, and sequences of CRF07_BC formed the largest HIV-1 transmission cluster, which contained 272 nodes. In addition, the transmission cluster derived from CRF07_BC contained 67 nodes at a genetic distance threshold of 0.5%, indicating the ongoing transmission of HIV CRF07_BC subtype. Univariate analysis showed significant differences in the proportion of sequences included in the HIV transmission molecular network in terms of age groups, HIV subtype, emergence of HIV drug resistance and marital status (*p* < 0.05). Multivariate logistic regression analysis showed that ages of under 20 years and over 60 years, CRF07_BC subtype, Han ethnicity, sampling in 2015, absence of HIV drug resistance, married with spouse, sampling from JJ, NP, and QZ resulted in higher proportions of sequences included in the HIV transmission molecular network (*p* < 0.05). Further studies to investigate the causes responsible for the high proportion of inclusion of sequences from HIV-infected individuals are needed for targeted interventions, so as to reduce HIV transmission and new infections.

There has been an increase in the number and proportion of newly reported HIV/AIDS cases among individuals at ages of 60 years and older in Fujian province from 2012 to 2022. Our findings showed the highest proportion of inclusion in the HIV molecular network among patients at ages of 60 years and older (55.7%), which is consistent with the epidemiological characteristics of HIV/AIDS in the province. Intensified epidemiological investigations and molecular network dynamic monitoring, timely identification of active transmission networks and high-risk carriers, and targeted interventions are required targeting these high-risk populations to reduce the HIV transmission risk and new infections.

The prevalence of HIV infection has been increasing in MSM over years (33). The prevalence of HIV-1 infection has been maintained at a high level among MSM in Fujian province. During the investigation and tracking of the transmission process of HIV-1 in MSM, conventional epidemiological tools based on questionnaire surveys and peer tracking may have various biases, resulting in low credibility of conclusions and subsequent unfavorable follow-up and behavioral interventions. Recently, HIV-1 molecular transmission networks using the viral gene sequences of infected individuals has gradually been employed to accurately identify potential transmission and determine active transmission networks, thus facilitating targeted intervention measures (34–38).

In this study, molecular network analysis showed more connections between the route of MSM and heterosexual transmission than the route of MSM, indicating the strong association between two routes of heterosexual transmission and MSM. in most of the transmission clusters, different routes of HIV transmission appear in the same network, and in different routes of HIV transmission, there is a “key person” who is a man who has sex with men but reports heterosexuality. During routine HIV/AIDS epidemiology surveys, data pertaining to sexual behaviors acquired may be inaccurate, which may affect the formulation of HIV/AIDS control measures. Therefore, by creating an HIV molecular network, we can find these errors and correct them through further investigations, so as to facilitate the understanding of the true epidemiological characteristics of HIV/AIDS in Fujian Province and provide insights into HIV/AIDS prevention and control.

In addition, the highest numbers of connections were observed between samples sites from Fuzhou city and Quanzhou city, indicating the important role of Fuzhou and Quanzhou cities in HIV transmission across Fujian province, which may be attributed to convenient transportation and geographical proximity.

The transmitted HIV drug resistance has shown a tendency toward a rise in Fujian province since 2008, and a more remarkable increase has been found since 2012. The overall prevalence of transmitted drug resistance was 4.4% among treatment-naive HIV-1-infected patients in Shaanxi province, northwestern China from 2003 to 2013, and the prevalence became stable between 2009 and 2013 (*p* = 0.982) (39). The overall prevalence of transmitted HIV drug resistance was 3.42% among newly diagnosed HIV-1 individuals in Jiangsu province, China during the period from 2009 through 2011 (40). In addition, the proportion of transmitted drug resistance was 4.4% among recently infected HIV-positive individuals in Hong Kong from 2007 to 2010 (41) and the prevalence of transmitted drug resistance of HIV-1 strains was 11.1% among individuals attending voluntary counseling and testing in Taiwan from 2006 to 2014 (42). In the current study, the overall prevalence of HIV drug resistance was 4.8%, and the prevalence was 4.2% in 2015 and 5.3% in 2020. In addition, molecular network analysis identified three transmission clusters (Cluster 11, 31 and 75), and these three clusters all carried E138G and V189E, which led to NNRTIs resistance. Epidemiological data showed that these three clusters were all formed by CRF55_01B subtype, and patients carrying these sequences were all men and predominantly single, indicating that the transmission of HIV drug resistance mainly occurs in MSM. Further studies to unravel the underlying mechanisms, screen high-risk population for HIV and implement targeted interventions are needed to avoid the spread of HIV drug resistance.

The current study has some limitations. First, HIV-uninfected individuals were not included. This study aimed to unravel the molecular transmission network of newly diagnosed HIV/AIDS patients in Fujian province, southeastern China. HIV molecular transmission network consists of a group of HIV-infected individuals with potential transmission relationships, and the HIV that individuals infect has a genetic similarity (43). Therefore, HIV-uninfected individuals at a high risk of infection were not recruited in the present study. Nevertheless, HIV-uninfected individuals at a potential risk of infection will be included in the creation of the HIV risk network based on field epidemiological surveys after construction of the HIV molecular network. Second, the molecular clusters generated from the HIV molecular network are only effective to identify the transmission relationship among individuals included in the molecular clusters, but fail to unravel the transmission of virus (44). Further field epidemiological surveys are required to identify potential HIV spreader.

In summary, we, for the first time, create the HIV molecular transmission network of newly diagnosed HIV/AIDS patients in Fujian province, southeastern China, which facilitates the understanding of HIV transmission patterns in the province. A comprehensive analysis of the transmission clusters in the HIV molecular network, demographics, epidemiological features and laboratory testing facilitates the formulation of targeted interventions for HIV/AIDS, so as to reduce new HIV infections.

## Data availability statement

The raw data supporting the conclusions of this article will be made available by the authors, without undue reservation.

## Ethics statement

The studies involving humans were approved by the Fujian Provincial Center for Disease Control and Prevention. The studies were conducted in accordance with the local legislation and institutional requirements. Written informed consent for participation in this study was provided by the participants’ legal guardians/next of kin.

## Author contributions

All authors listed have made a substantial, direct, and intellectual contribution to the work and approved it for publication.
